# A Novel Behavioral Assay for Measuring Cold Sensation in Mice

**DOI:** 10.1371/journal.pone.0039765

**Published:** 2012-06-22

**Authors:** Daniel S. Brenner, Judith P. Golden, Robert W. Gereau

**Affiliations:** 1 Washington University Pain Center and Department of Anesthesiology, Washington University School of Medicine, St. Louis, Missouri, United States of America; 2 Neuroscience Program, Washington University School of Medicine, St. Louis, Missouri, United States of America; 3 Medical Scientist Training Program, Washington University School of Medicine, St. Louis, Missouri, United States of America; Tokai University, Japan

## Abstract

Behavioral models of cold responses are important tools for exploring the molecular mechanisms of cold sensation. To complement the currently cold behavioral assays and allow further studies of these mechanisms, we have developed a new technique to measure the cold response threshold, the cold plantar assay. In this assay, animals are acclimated on a glass plate and a cold stimulus is applied to the hindpaw through the glass using a pellet of compressed dry ice. The latency to withdrawal from the cooled glass is used as a measure of the cold response threshold of the rodents, and the dry ice pellet provides a ramping cold stimulus on the glass that allows the correlation of withdrawal latency values to rough estimates of the cold response threshold temperature. The assay is highly sensitive to manipulations including morphine-induced analgesia, Complete Freund's Adjuvant-induced inflammatory allodynia, and Spinal Nerve Ligation-induced neuropathic allodynia.

## Introduction

In every field of medicine, pain is a significant issue [1], [2]. One clinically important subset of pain is aberrant cold perception, which affects broad patient populations including multiple sclerosis [3], chemotherapy [4], [5], [6], and stroke patients [7], [8]. These patients experience alterations in cold sensitivity that profoundly impact their quality of life, including alterations of the cold response threshold (cold allodynia) and of the severity of the evoked sensation (cold hyperalgesia)[3], [7], [9], [10]. Treatment options for these patients are limited, in part because the pathologic changes leading to alterations in cold sensitivity are poorly understood. The ability to treat these conditions should improve as the molecular mechanisms of cold pain and sensitization become better understood. Progress in understanding these mechanisms has been slowed by limitations in the behavioral assays used to study animal models of aberrant cold perception, suggesting that new, complementary assays are necessary.

One common model, the acetone evaporation test [11], [12], involves dabbing the hindpaw with acetone, which evaporates and cools the paw. The time the mouse spends flicking the paw afterwards is used as a measure of cold sensitivity. While this method has been a mainstay of investigations and has led to important discoveries, there are several limitations. The test is confounded by the odor of acetone on the paw, and naive mice have no response, which limits the test's utility in measuring cold anesthesia. Additionally, while the acetone cooling has been quantified [13], it is difficult to ensure that the exact same amount of acetone is applied to generate a consistent stimulus, and impossible to modulate the rate of cooling. Additionally, this assay quantifies the magnitude of responses after the application of a cold stimulus, as opposed to the minimal cold temperature capable of generating a response (the cold response threshold).

Another commonly used test is the tail flick assay, in which the latency of mice to withdraw their tails from cold water is measured [12], [14]. While the behavioral response is unambiguous, the animals must be restrained during testing, which causes stress and may alter results through well–known stress-induced analgesic mechanisms [15].

Another model, the cold plate assay, assesses the behavior of mice after they are placed on a pre-cooled plate [16], [17], [18]. Investigators have used metrics including the number of jumps [13], [16], the latency to first response [13], [16], [19], [20], and the number of paw lifts [13], [21], [22], but there is no consensus on the meaning of each parameter. Furthermore, only one temperature can be tested at a time. As with the acetone test, the cold plate does not assess a precise temperature capable of generating a response, but measures the magnitude of the response.

A related technique, the 2 temperature choice test, measures the preference of an animal between two temperature-controlled plates, typically a control temperature and a cold temperature. The time spent on each plate is thought to reflect the animal's temperature preferences over a long period, as opposed to the spinal reflexes measured in the assays described above. One limitation of this assay is that the data reflects preference rather than aversion, and is therefore difficult to interpret physiologically. It is also time and manpower intensive, requiring multiple trials at every temperature to generate a full temperature response curve.

To complement the currently used assays and accurately measure cold response threshold, we developed a novel technique, the cold plantar assay. Mice are acclimated on glass, a pellet of compressed dry ice powder is applied to the glass below the paw, and the latency to withdrawal from the cooling glass is measured. We demonstrate that this method delivers a consistent ramping cold stimulus to acclimated, unrestrained mice.

## Methods

### Animals

All mouse protocols were in accordance with National Institutes of Health guidelines and were approved by the Animal Studies Committee of Washington University School of Medicine (St. Louis, MO). All experiments were carried out with 6–8 week old male Swiss Webster mice (Jackson Labs, Bar Harbor, ME) except for 6–8 week old male C57/Bl6 mice used in Figure 2B, which were bred in-house. Mice were housed on a 12/12 hour light/dark cycle with the light cycle beginning at 6 a.m. All mice had *ad libitum* access to rodent chow and water.

### Data analysis

All data were collated in.xlsx files using Microsoft Excel 2011 and analyzed using Graphpad Prism from Graphpad Software (La Jolla, CA). Unless otherwise noted, all error bars shown represent the standard error of the mean.

### Behavioral analysis

All behavioral tests and analyses were conducted by an experimenter blind to treatment. Behavioral tests were performed between 1pm and 5 p.m. local time unless the experimental design required baseline measurements in the morning (CFA injection only). All experiments were performed at 22°C.

### Cold plantar assay

1/8″, 3/16″, and 1/4″ thick pyrex borosilicate float glass was acquired from Stemmerich, Inc (St. Louis, MO). Mice were acclimated on the glass plate in transparent plastic enclosures (4″×4″×11″) separated by opaque black dividers for 2.5–3 hours. The lighting was undimmed to maintain the light/dark cycle and a white noise generator was used to mask external noise.

**Figure 1:**
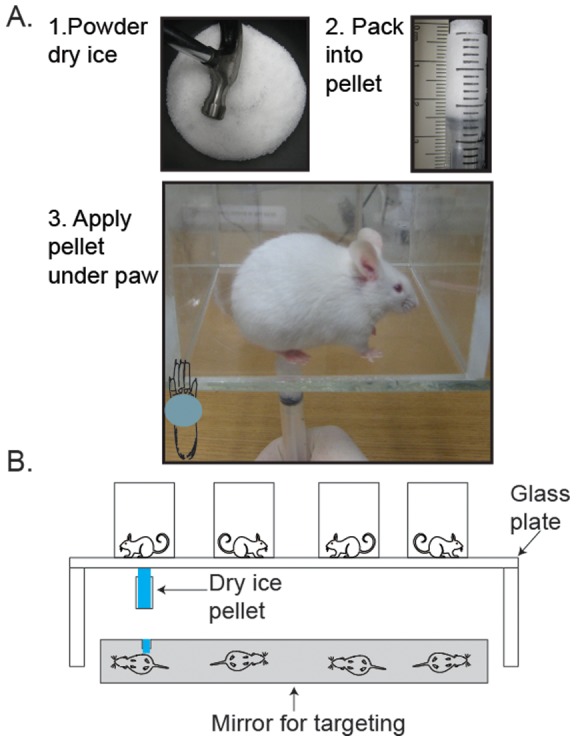
The Cold Plantar Assay. A. Grind dry ice into a fine powder with a hammer (panel 1). Load the dry ice powder into a 3mL syringe and compress against a flat surface until powder cannot be compressed any further (panel 2). Using the syringe plunger, push the dry ice pellet 20–30mm past the tip of the syringe (panel 2). Gently but firmly apply the flat end of the dry ice pellet to the glass surface underneath the paw of the mouse (panel 3) and measure the latency to withdrawal with a stopwatch. B. Schematic of the cold plantar assay apparatus. Mice are acclimated in plastic enclosures on a glass plate. A mirror is placed underneath the apparatus to facilitate targeting of the dry ice probe.

To make the cold probe, freshly delivered dry ice was crushed into a fine powder using a hammer and stored at −80°C. To shape the probe, a razor blade was used to cut the top off a 3mL BD syringe (Franklin Lakes, NJ), and a BD needle tip was used to make 3 holes on each side of the syringe to prevent gas buildup inside the syringe body. The powdered dry ice was packed into the modified syringe and the open end of the syringe was held against a flat surface while pressure was applied to the plunger to compress the dry ice into a flattened, dense pellet 1 cm in diameter (Figure 1).

**Figure 2:**
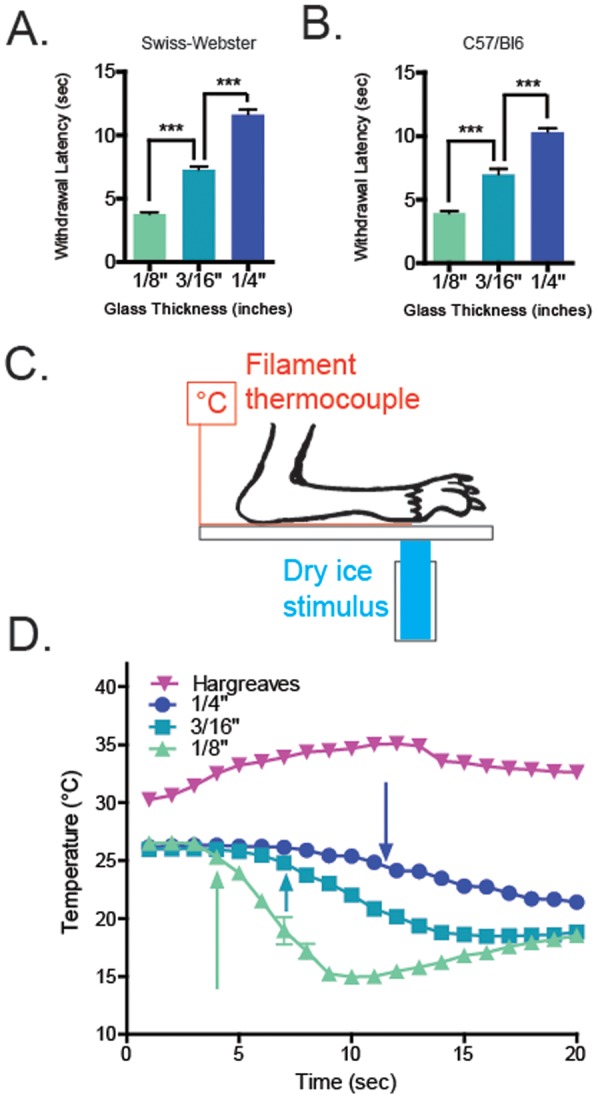
The Cold plantar assay applies a consistent ramping cold stimulus. A. The cold plantar withdrawal latency increases as thicker glass plates are used. With Swiss Webster mice, on the 1/8″ glass the average latency is 3.79±0.5 seconds, on the 3/16″ glass the average latency is 7.29±0.9 seconds, and on the 1/4″ glass the average latency is 11.63±1.3 seconds. The average latencies on each glass are significantly different (1-way ANOVA with Bonferroni post-hoc test ***p<.0001 between all thicknesses; n = 12 mice per glass). B. With C57 mice, on the 1/8″ glass the average latency is 3.83±0.5 seconds, on the 3/16″ glass the average latency is 7.0±1.4 seconds, and on the 1/4″ glass the average latency is 10.4±0.9 seconds. The average latencies on each glass are significantly different (1-way ANOVA with Bonferroni post-hoc test ***p<.0001 between all thicknesses; n = 10 mice per glass). C. Schematic showing how the temperature between the paw and the glass was measured. D. Curves representing the change in temperature between the paw and the glass during and shortly after dry ice stimulation. For each curve, the dry ice stimulus begins at x = 1, and ends at the colored arrow for that thickness (from 2A, 1/8″: 3.79 seconds, 3/16″: 7.29 seconds, 1/4″: 11.63 seconds). Based on these curves, mice withdraw after a 1.3°C (1/8″), 1.5°C (3/16″), or 2°C (1/4″) change in temperature (n = 6 per glass thickness).

**Figure 3:**
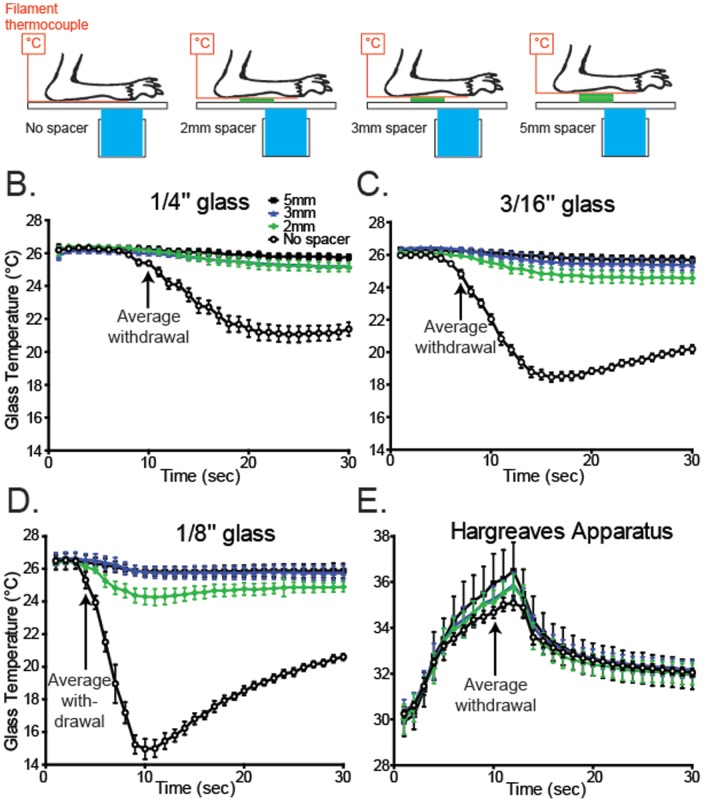
The cold plantar assay requires direct paw-glass contact. A. Schematic diagramming the experimental design. B–D. The temperature during cold plantar stimulus underneath the paw was measured on all three glass thicknesses under normal conditions, and with styrofoam spacers propping the paw away from the glass surface. In all cases, propping the paw away from the glass caused a dramatic decrease in the cold stimulus measured at the paw (n = 6 per glass thickness). E. The temperature underneath the paw was measured during Hargreaves radiant heat stimulation with the paw propped up with styrofoam. Unlike the cold plantar assay, the thermal stimulus in the Hargreaves assay is largely unaltered when the paw is propped away from the glass (n = 6).

**Figure 4:**
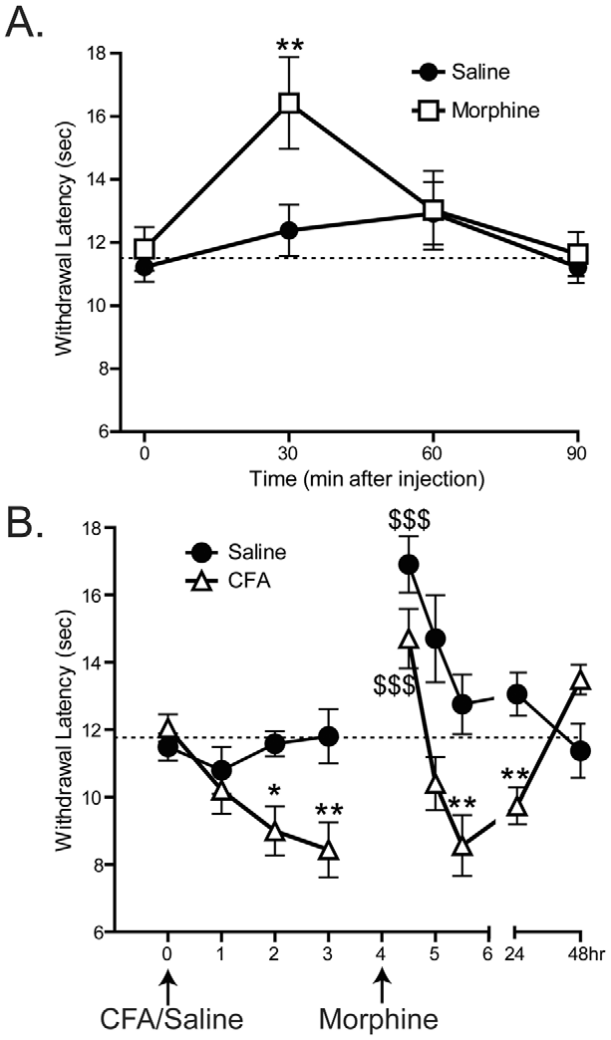
The cold plantar assay can measure analgesia and allodynia. A. Mice were given subcutaneous injections of 1.5mg/kg morphine or saline. Thirty minutes after injection, morphine-injected mice had significantly higher withdrawal latencies on the 1/4″ glass, but 60 minutes after injection the morphine injected mice had returned to baseline latency values (2-way ANOVA main effect *p<0.05 with Bonferroni post-hoc test; 30 minutes **p<0.01; n = 12 per group). B. Mice were given intraplantar injections of Complete Freund's Adjuvant or saline. 2 and 3 hours after injection, CFA-injected mice had significantly lower withdrawal latencies on the 1/4″ glass than the saline-injected controls (2-way ANOVA main effect p<.0001 with Bonferroni post-hoc test; 120 minutes *p<0.05, 180 minutes **p<0.01 n = 12 per group). 4 hours after intraplantar injection, all animals were given subcutaneous injections of 1.5mg/kg morphine. At 4.5 hours both CFA- and Saline-injected animals had significantly increased withdrawal latencies compared to their values at 3 hours (1-way ANOVA with Dunnett's post-hoc test; CFA 3hours vs. CFA 4.5 hours $$$ p<0.0001, Saline 3 hours vs. Saline 4.5 hours $$$ p<0.0001). At 5.5 hours, the CFA-injected mice had significantly lower withdrawal latencies than their saline-injected counterparts (2-way ANOVA with Bonferroni post-hoc test; 5.5 hours **p<0.01). 24 hours after intraplantar injection, the CFA-injected mice still had significantly lower withdrawal latencies than their saline-injected counterparts, but this difference resolved by 48 hours (2-way ANOVA with Bonferroni post-hoc test; 24 hours ** p<0.01, 48 hours p>0.05).

**Figure 5:**
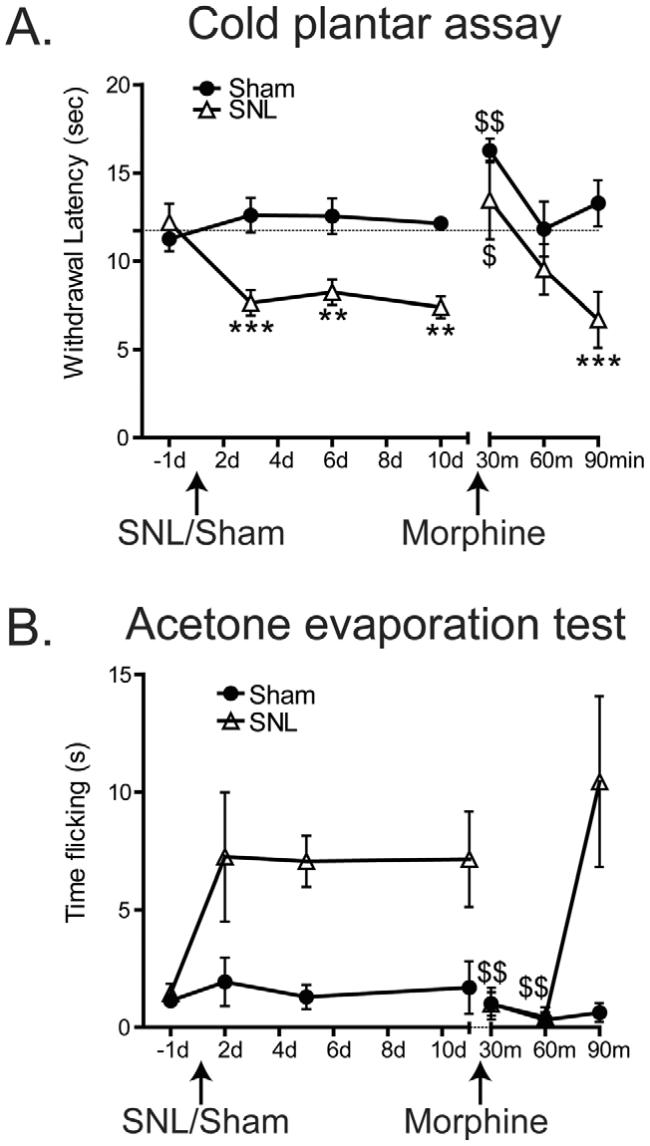
The cold plantar assay can measure L4 spinal nerve ligation induced allodynia. Mice underwent the spinal nerve ligation (SNL) procedure or a sham procedure on day 0. A. On days 3, 6, and 10 post surgery SNL mice had significantly lower cold plantar withdrawal latencies on the 1/4″ glass than sham mice (2-way ANOVA main effect ***p<0.001 with Bonferroni post-hoc test; 3d ***p<0.001, 6d **p<0.01, 10d **p<0.01; Sham n = 5, SNL n = 6). After baseline measurements on day 10 post surgery, mice were injected with 1.5mg/kg morphine. Thirty minutes after morphine injection, both SNL and sham mice had withdrawal latencies that were significantly elevated from their baseline values that day (1-way ANOVA with Dunnett's post-hoc test; Sham $ p<0.05, SNL $$ p<0.01). By 90 minutes after morphine injection, the SNL mice again had significantly decreased withdrawal latencies than the sham mice (2-way ANOVA with Bonferroni post-hoc test; ***p<0.001). B. On days 2, 5, and 11 post surgery SNL mice spent more time flicking after acetone application than sham mice (2-way ANOVA main effect *p<0.05 with Bonferroni post-hoc test; 2 days p>0.05, 5 days p>0.05, 11 days p>0.05). After baseline measurements on day 11 post surgery, mice were injected with 1.5mg/kg morphine. There was a significant reduction in the time responding to acetone in the SNL group but not in the Sham group (1-way ANOVA with Dunnett's post-hoc test; Sham p>0.05, SNL $$ p<0.01).

Mice at rest but not asleep were tested by extending the tip of the dry ice pellet past the end of the syringe and pressing it to the glass underneath the hindpaw using light pressure but consistent pressure applied to the syringe plunger (**Figure** **1**). The center of the hindpaw was targeted, taking care to avoid the distal joints and ensuring that the paw itself was actually touching the glass surface (**Figure** **1**).

The withdrawal latency was measured with a stopwatch. Withdrawal was defined as any action to move the paw vertically or horizontally away from the cold glass. An interval of at least 7 minutes was allowed between testing separate paws on a single mouse, and an interval of at least 15 minutes was allowed between trials on any single paw. These intervals were chosen empirically to allow enough time for the average mouse to return to a resting state after stimulation. For baseline experiments, mice were measured at least 3 times per paw. For experiments with an experimentally limited time window, 1–2 measurements per paw were made per timepoint.

The maximum time allowed for withdrawal was 20 seconds to avoid potential tissue damage, based on animal behavior in preliminary trials; in early tests, mice that endured longer that 20 second stimulations responded with exaggerated nocifensive behaviors and would often prevent additional testing on that paw by not placing it on the glass. Trials where the animal did not withdraw in under 20 seconds were repeated. If the second trial also yielded no withdrawal within the cutoff, the value was recorded as 20 seconds.

### Acetone evaporation assay

The acetone evaporation assay was performed as described previously [23]. Briefly, mice were acclimated for 3 hours on a wire mesh. After acclimation, acetone was drawn into a 1mL syringe and dabbed onto the plantar surface of the hindpaw. The first 10 seconds of activity were disregarded as a response to the direct application of the droplet [23], and the time spent flicking or licking the paw for 60 seconds afterwards was measured with a stopwatch.

### Glass temperature measurements

Temperature values on the glass surface were measured using an IT-24P filament T-type thermocouple probe from Physitemp Instruments, Inc. (Clifton, NJ) and the temperatures were collected once every second using an EA15 Data-Logging Dual Input Thermometer from Extech Instruments (Waltham, MA). The data were output into CSV files which were loaded into Microsoft Excel.xlsx files and analyzed using Graphpad Prism from Graphpad Software (La Jolla, CA).

To measure temperature readings on the glass, Swiss Webster mice were lightly anesthetized using a cocktail of Ketamine (final concentration 38 mg/mL Fort Dodge Animal Health, Fort Dodge IA), Xylazine (final concentration 1.92 mg/mL Lloyd Labs Shenandoah, IA) Acepromazine (final concentration 0.38 mg/mL Butler Schein Animal Health Dublin, OH). The T-type thermocouple filament was secured to the mouse paw with a loop of laboratory tape. The paw was then taped to the glass and gently held flush to the glass surface. A dry ice pellet was generated as described above and held to the underside of the glass in the target area for a proscribed amount of time and the change in temperature was tracked with the EA15 Data Logger. This analysis was performed on each of the 3 thicknesses of glass (1/8″, 3/16″, 1/4″). Temperature measurements were also performed using a Hargreaves glass apparatus from IITC Life Sciences (Woodland Hills, CA) where the active intensity was engaged as soon as the light was focused onto the paw to avoid premature warming from the resting intensity beam. The light stimulus was applied for the average withdrawal latency with the active intensity of 15% (10 seconds).

### Morphine studies

Morphine sulfate from Baxter Healthcare (Deerfield, IL) was diluted in saline and injected subcutaneously at a final dosage of 1.5mg/kg to induce short-term anesthesia. Mice were injected with either morphine or saline vehicle, and the experimenter was blind to the contents of each syringe until after data analysis. No mice were excluded from analysis. Mice were acclimated as above, and 3 baseline measurements of withdrawal latency to cold stimulus for the right paw were taken for each mouse. The mice were then injected subcutaneously with either the morphine or a saline control and measurements of withdrawal latency were taken from the right paw of each animal every 30 minutes until all mice returned to baseline latency values.

### Complete Freund's Adjuvant (CFA) studies

Twenty microliters of Complete Freund's Adjuvant from Sigma-Aldrich (St. Louis, MO) or 0.9% Saline from Hospira, Inc (Lake Forest, IL) was injected into the right hindpaw of each mouse. The experimenter was blind to the contents of each syringe until after data analysis was completed. No mice were excluded from analysis. Mice were acclimated as described above and 3–5 baseline measurements of withdrawal latency for each paw were taken for each mouse. Each mouse was then injected in the right hindpaw with either CFA or saline and measurements of cold plantar withdrawal latency on both paws were measured at 1 hour, 2 hours, and 3 hours post-injection. At 4 hours post intraplantar injection, all mice were given subcutaneous injections of 1.5mg/kg morphine and the withdrawal latency was measured at 4.5, 5, and 5.5 hours post intraplantar injection. Finally, withdrawal latency measurements were also made at 24 and 48 hours post-intraplantar injection.

### Spinal nerve ligation (SNL) model

The spinal nerve ligation procedure was performed as described previously [24], [25]. The experimenter was blinded to the surgical procedure received by each mouse until after data analysis was completed. No mice were excluded from analysis. Briefly, baseline measurements of cold plantar withdrawal latency and acetone evaporation response time were made on all mice. The mice were then deeply anesthetized with the ketamine cocktail described above, and the paraspinal muscles were bluntly dissected to expose the left L5 transverse process. Mice receiving the full ligation procedure had the L5 process removed, and the left L4 spinal nerve was tightly ligated with silk suture (6-0, Ethicon; Cornelia, GA) and transected distal to the ligation. Mice receiving a sham surgery had the L5 transverse process exposed, but not removed and the nerve was untouched.

The cold plantar withdrawal latency was then measured 3, 6, and 10 days after surgery. The time spent responding to acetone evaporation was measured 2, 5, and 11 days after surgery. After the baseline measurements on days 10/11, on both days the mice were injected subcutaneously with 1.5mg/kg morphine and either cold plantar withdrawal threshold or acetone response time was measured 30, 60, and 90 minutes after morphine injection.

## Results

### Mice respond to consistent changes in temperature in the cold plantar assay

To measure the cold response threshold of mice to a cold stimulus, we apply a compressed dry ice pellet underneath the hindpaw as it rests on a glass plate as depicted in **Figure** **1**. Condensation generally appeared on the glass after stimulation, but not during. Application of the dry ice pellet resulted in a number of nocifensive responses. Typical responses from mice at rest but not asleep included lifting of the paw followed by a combination of flicking, licking, or biting the paw lasting 1 second to one minute after cessation of the stimulus and/or movement away from the stimulus area. The latency to withdrawal from the cooled glass is used as a surrogate for the temperature at which the mice responded to the cold stimulus. This latency is dependent on the thickness of the glass plate. We measured the cold threshold response in naïve Swiss Webster mice on 1/8″, 3/16″, and 1/4″ thick glass. Latency for a mouse to remove its hindpaw from the glass surface was lowest for the 1/8″ plate (3.79±0.5 seconds), and increased as the thickness increased to 3/16″ (7.29±0.9 seconds) and then 1/4″ (11.63±1.3 seconds) (**Figure** **2A** 1-way ANOVA with Bonferroni post-hoc test ***p<0.0001 for all comparisons; n =  12 per glass). The average cold plantar withdrawal latency of C57/Bl6 mice on all three thicknesses was also measured **(** **Figure** **2B** 1/8″ plate average: 3.83±0.5s, 3/16″ plate average: 7.0±1.4s, 1/4″ plate average: 10.4±0.9s 1-way ANOVA with Bonferroni post-hoc test ***p<0.0001 for all comparisons).

After establishing consistent withdrawal latencies on each glass thickness, we anesthetized mice and positioned their paws over a filament thermocouple probe on the glass (**Figure** **2B**) to measure the temperature between the paw and the glass during the cold stimulus used in this. To mimic experimental conditions, the cold stimulus was applied for the average latency to withdrawal for each thickness. In other words, we applied the stimulus for 4 seconds for 1/8″ thick glass (**Figure** **2C** **, light blue triangles, arrow at 4** **seconds)**, 7 seconds for 3/16″ thick glass (**Figure** **2C** **, royal blue squares, arrow at 7** **seconds)**, and 10 seconds for 1/4″ thick glass (**Figure** **2C** **, dark blue circles, arrow at 10** **seconds)**. On the 1/8″ glass, the temperature between paw and glass after 4 seconds of stimulation was decreased by 1.3°C. (**Figure** **2C** n = 6). On the 3/16″ thick glass, the temperature between the paw and the glass after 7 seconds of stimulation was decreased by 1.5°C (**Figure** **2C** n = 6). On the 1/4″ thick glass, the temperature between the paw and the glass after 10 seconds of stimulation was decreased by 2°C (**Figure** **2C** n = 6). When the dry ice stimulus is maintained on the glass plates past these average withdrawal times, the temperature continues to decrease rapidly and can reach temperatures below −5°C (data not shown). We also performed this temperature measurement analysis using the conventional radiant heat apparatus (IITC; Woodland Hills, CA) with a pre-heated glass plate, and found that after 10 seconds of radiant thermal stimulation (the average stimulus capable of causing withdrawal under these conditions) the temperature between the glass and the paw increased by 4.3°C (**Figure** **2C** n = 6).

Next we tested whether direct contact of the paw with the glass surface is required to generate a consistent cold stimulus. To test this, we used thin styrofoam spacers to incrementally separate the paw and thermocouple from the glass surface while applying the cold stimulus (**Figure** **3A** n = 6 per group). When the paw is separated from the glass surface, the cold stimulus from the dry ice pellet is drastically attenuated (**Figure** **3B–D**). We also performed this analysis using the Hargreaves apparatus on a pre-heated plate, and found that the radiant heat stimulus was unaffected by the separation of the paw from the glass (**Figure** **3E**).

### Morphine-induced anesthesia

After 3–5 baseline measurements of cold plantar withdrawal latency were made on each animal, transient anesthesia was induced with subcutaneous injections of 1.5mg/kg morphine over the coccyx. Cold plantar withdrawal latency measurements on the 1/4″ glass were taken on the right hindpaw at 30, 60, and 90 minutes after injection of the morphine or a saline control. All testing and analysis was done while blinded to treatment. The latency to withdraw was significantly increased after 30 minutes for the morphine-injected group compared to the saline-injected group (**Figure** **4A** 2-way ANOVA main effect *p<0.05 with Bonferroni post-hoc test; 30 minutes **p<0.01; n = 12 per group). By 60 minutes post-injection the latency to withdraw returned to baseline levels (**Figure** **4A** 2-way ANOVA with Bonferroni post-hoc test; 60 minutes p>0.05), consistent with the typical rate of morphine metabolism in mice [26].

### Complete Freund's Adjuvant (CFA)-induced hypersensitivity

After 3–5 baseline measurements of cold plantar withdrawal latency were made on each animal, inflammation was induced with intraplantar injections of 20μL CFA. Using the 1/4″ glass, the cold plantar withdrawal latency was measured on both hindpaws (injected and contralateral) at 1, 2, and 3 hours after injection with CFA or a saline control. 4 hours after CFA injection, all mice were injected with 1.5mg/kg morphine and withdrawal latencies were measured at 4.5, 5, and 5.5 hours. Over the following two days, paw withdrawal latencies were measured 24 and 48 hours after CFA injection. After 120 and 180 minutes, the latency to withdraw significantly decreased in the mice injected with CFA compared to mice injected with saline (**Figure** **4B** 2-way ANOVA main effect p<.0001 with Bonferroni post-hoc test; 120 minutes *p<0.05, 180 minutes **p<0.01 n =  12 per group). At 4.5 hours, 30 minutes after the subcutaneous injection of morphine, the latency to withdraw of both groups of mice increased significantly compared with their respective latencies before the morphine injection (**Figure** **4B** 1-way ANOVA with Dunnett's post-hoc test; CFA 3 hours vs. CFA 4.5 hours $$$p<0.0001, Saline 3 hours vs. Saline 4.5 hours $$$p<0.0001). At 5 hours after intraplantar injections (60 minutes after morphine injection), neither group was significantly different from the withdrawal latencies at 3 hours (**Figure** **4B** 1-way ANOVA with Dunnett's post-hoc test; CFA and Saline p>0.05). By 5.5 hours after intraplantar injection (90 minutes after morphine injection), the withdrawal latencies of the mice injected with CFA were significantly lower than those of mice injected with saline (**Figure** **4B** 2-way ANOVA with Bonferroni post-hoc test; 5.5 hours **p<0.01). The cold hypersensitivity that was induced by CFA injection was maintained for 24 hours (**Figure** **4B** 2-way ANOVA with Bonferroni post-hoc test; CFA ** p<0.01), but returned to the same values as the saline-injected mice by 48 hours (**Figure** **4B** 2-way ANOVA with Bonferroni post-hoc test; p>0.05). Contralateral paw withdrawal latencies were measured at all time points, and were unchanged by experimental manipulations (data not shown).

### Spinal nerve ligation-induced hypersensitivity

After 3–5 baseline measurements of cold plantar withdrawal latency using the 1/4″ thick glass as well as the acetone evaporation test were made on each animal, nerve injury was induced using the left L4 spinal nerve ligation (SNL) procedure [24], [25] on 6 week-old male Swiss Webster mice (**Figure** **5**). After acetone application the amount of time spent flicking was significantly increased in the SNL mice compared to the sham mice, but no individual time points showed increases at 2, 5, or 11 days after surgery showed increases that were statistically significant in post-hoc tests (**Figure** **5B** 2-way ANOVA main effect *p<0.05 with Bonferroni post-hoc test; 2 days p>0.05, 5 days p>0.05, 11 days p>0.05; Sham n =  5, SNL n =  6). When tested with the cold plantar assay, the same SNL mice yielded a significant main effect of SNL treatment leading to lower withdrawal latencies, and also had decreases in latencies at 3, 6, and 10 days after surgery that were statistically significant by post-hoc tests (**Figure** **5A** 2-way ANOVA main effect ***p<0.001 with Bonferroni post-hoc test; 3 days ***p<0.001, 6 days **p<0.01, 10 days **p<0.01).

Ten days after surgery, cold plantar withdrawal latencies were measured and then 1.5mg/kg morphine was injected subcutaneously. Cold plantar assay measurements were taken at 30, 60, and 90 minutes after injection. Both sham and SNL groups showed increased withdrawal latencies 30 minutes after morphine injection (**Figure** **5A** 1-way ANOVA with Dunnett's post-hoc test; Sham $$p<0.05, CFA $p<0.05), and both groups returned to their respective baseline values by 60 minutes after morphine (**Figure** **5A** 2-way ANOVA with Bonferroni post-hoc test; ***p<0.001).

Eleven days after surgery, acetone evaporation baselines were measured and then 1.5mg/kg morphine was injected subcutaneously. Acetone evaporation measurements were taken at 30, 60, and 90 minutes after injection. There was a significant reduction in the time responding to acetone in the SNL group but not in the Sham group (**Figure** **5B** 1-way ANOVA with Dunnett's post-hoc test; SNL 30 minutes and 60 minutes $$p<0.05, Sham p>0.05). Contralateral paw withdrawal latencies were measured at all time points, and were unchanged by experimental manipulations (data not shown).

## Discussion

We have demonstrated that the cold plantar assay allows the application of a consistent focal ramping cold stimulus to unrestrained, acclimated mice. This assay can measure both cold allodynia and cold anesthesia under baseline conditions with relatively low variability in mice in a way that was not possible using previously available techniques, such as the acetone evaporation test. It is also highly sensitive to procedures that alter the cold responses of mice including morphine, CFA-induced inflammation, and SNL-induced nerve injury.

In the cold plantar assay, we found that the paw being tested had to be in direct contact with the glass surface to maintain a consistent cold stimulus. Interestingly enough, our parallel studies with the radiant heat assay show that it does not share this requirement, as the stimulus delivered to the paw is largely unchanged whether the paw is in contact with the glass or not. The requirement for direct contact of the paw with the glass in the cold plantar assay is not an issue as acclimated mice typically rest with their paws on the glass. While testers should be wary of guarding or changes in weight distribution that may occur after injury, we have found that consistent measurements can be made even in both inflammatory and nerve-injury models (**Figures** **4** **,** **5**).

It was surprising to note that naïve mice withdraw from temperature decreases ranging between 1.3–2°C. Furthermore, our data using the Hargreaves apparatus also show that naïve mice withdraw after an increase of just 4–5°C, which is much smaller than previously reported values [27]. Based on these data, we conclude that a relatively small change in temperature is sufficient to generate a nocifensive response. That such small temperature changes elicit robust behavioral responses may suggest the nociceptive system is capable of responding to non-noxious temperature changes, or that withdrawal can be elicited by mechanisms that anticipate the potential severity of future insults and responds to prevent serious damage. This latter possibility is supported by several classical studies showing that the heat pain threshold is affected by the rate of heating [28], [29], which suggests the possibility that cold pain thresholds follow a similar principle. This notion is also supported by single unit recordings from cutaneous nociceptors showing that while nociceptive neurons fire bursts during cooling ramps, the firing rate decreases when the skin is held at a cold temperature for sustained periods [30], [31].

The molecular mechanisms of cold sensation and nociception have been hotly debated over the last decade, most notably over the roles of TRPM8 and TRPA1. Initial studies [17], [20] using the acetone evaporation test, cold plate test, and the 2 temperature choice tests showed no role for TRPA1 in cold sensation. However, studies [16], [22] published nearly simultaneously by other groups used the same assays and found that TRPA1 has a significant role in cold sensation. Part of the conflict may arise from ambiguities in interpreting the data from these assays; for example, in the cold plate test the one group measured the number of flinches [17], [20] but another group measured the number of paw lifts [22] and a third measured the latency to jump and the number of jumps [16]. The cold plantar assay, which yields an easily quantified and unambiguous behavioral response, will complement the previously available techniques and aid in addressing these controversies. This new technique will allow the consolidation of the work of the last decade, and enable further progress in understanding and manipulating the molecular mechanisms that underlie cold nociception, allowing for the development of new, targeted therapeutic agents.
